# Supplementary material for: Comparison of the Sensitivity and Specificity of Commercial Anti-Dengue Virus IgG Tests to Identify Persons Eligible for Dengue Vaccination
Source: medRxiv. 2024 Apr 20:2024.04.19.24306097. Preprint. [Version 1] doi: 10.1101/2024.04.19.24306097 (PMC11071579; doi:10.1101/2024.04.19.24306097)
Supplement: 1 [file NIHPP2024.04.19.24306097V1-supplement-1.pdf]

medRxiv preprint doi: https://doi.org/10.1101/2024.04.19.24306097; this version posted April 21, 2024. The copyright holder for this preprint (which was not certified by peer review) is the author/funder, who has granted medRxiv a license to display the preprint in perpetuity. This article is a US Government work. It is not subject to copyright under 17 USC 105 and is also made available for use under a CC0 license.

Figure S1. Sera from healthy children (ages 9-15) (n=400) living in Puerto Rico were tested in a focus reduction neutralization test and 50% virus neutralization titers (FRNT50) for DENV-1 (A), DENV-2 (B), DENV-3 (C), DENV-4 (D) and ZIKV (E) were determined as described in materials and shown according to immune status classification.

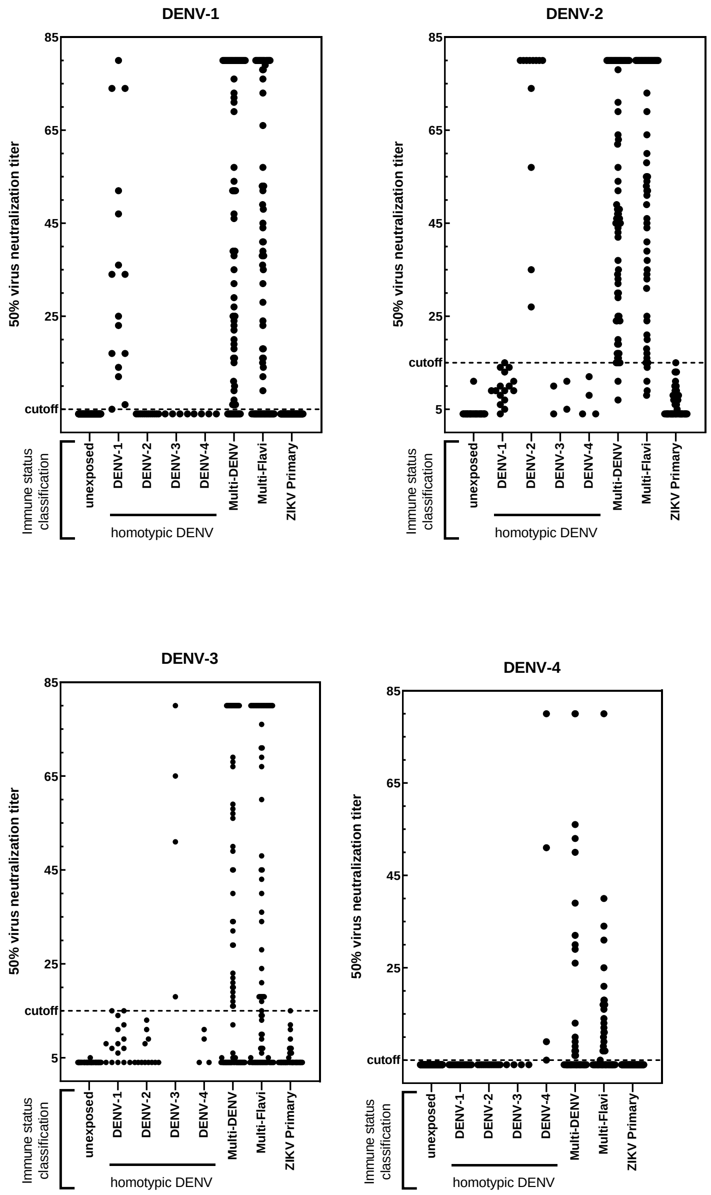

medRxiv preprint doi: https://doi.org/10.1101/2024.04.19.24306097; this version posted April 21, 2024. The copyright holder for this preprint
(which was not certified by peer review) is the author/funder, who has granted medRxiv a license to display the preprint in perpetuity.
This article is a US Government work. It is not subject to copyright under 17 USC 105 and is also made available
for use under a CC0 license.

Figure S2. STARD flow diagram for Euroimmun anti-DENV Type 1-4 ELISA IgG ELISA

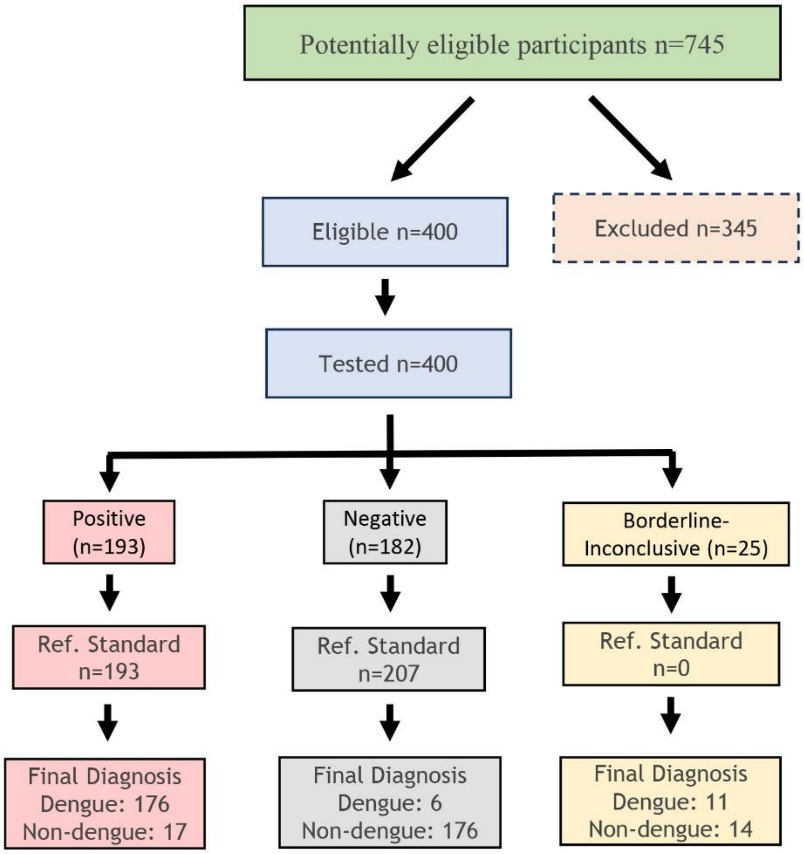

688

medRxiv preprint doi: https://doi.org/10.1101/2024.04.19.24306097; this version posted April 21, 2024. The copyright holder for this preprint (which was not certified by peer review) is the author/funder, who has granted medRxiv a license to display the preprint in perpetuity.
This article is a US Government work. It is not subject to copyright under 17 USC 105 and is also made available for use under a CC0 license.

Figure S3. STARD flow diagram for CTK OnSite Dengue IgG Rapid Test R0065C visual read

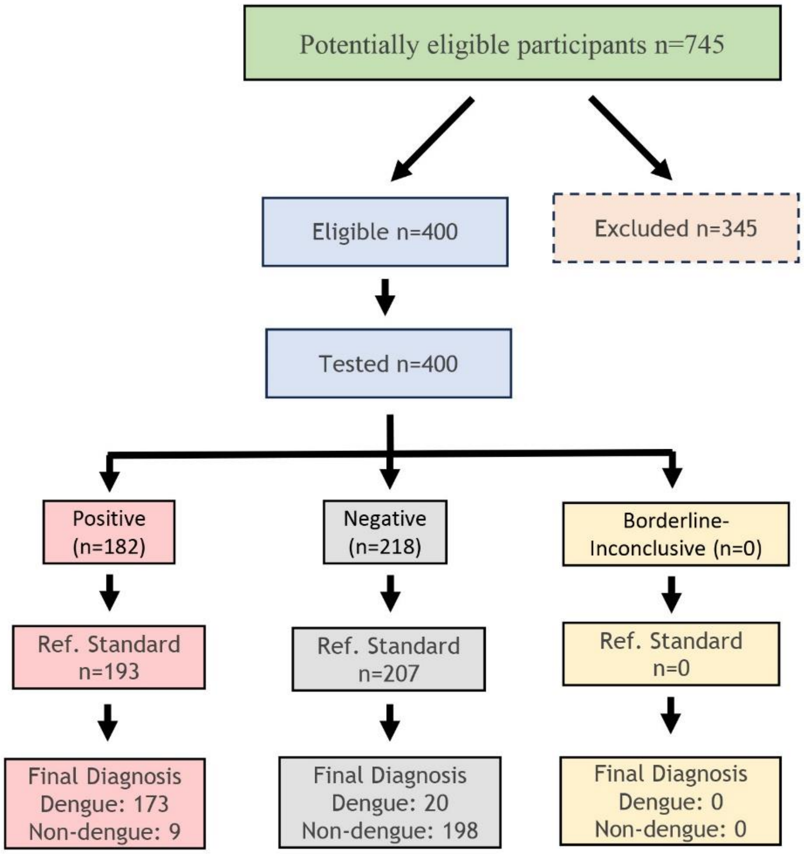

689

medRxiv preprint doi: https://doi.org/10.1101/2024.04.19.24306097; this version posted April 21, 2024. The copyright holder for this preprint
(which was not certified by peer review) is the author/funder, who has granted medRxiv a license to display the preprint in perpetuity.
This article is a US Government work. It is not subject to copyright under 17 USC 105 and is also made available
for use under a CC0 license.

Figure S4. STARD flow diagram for CTK OnSite Dengue IgG Rapid Test R0065C equipment read

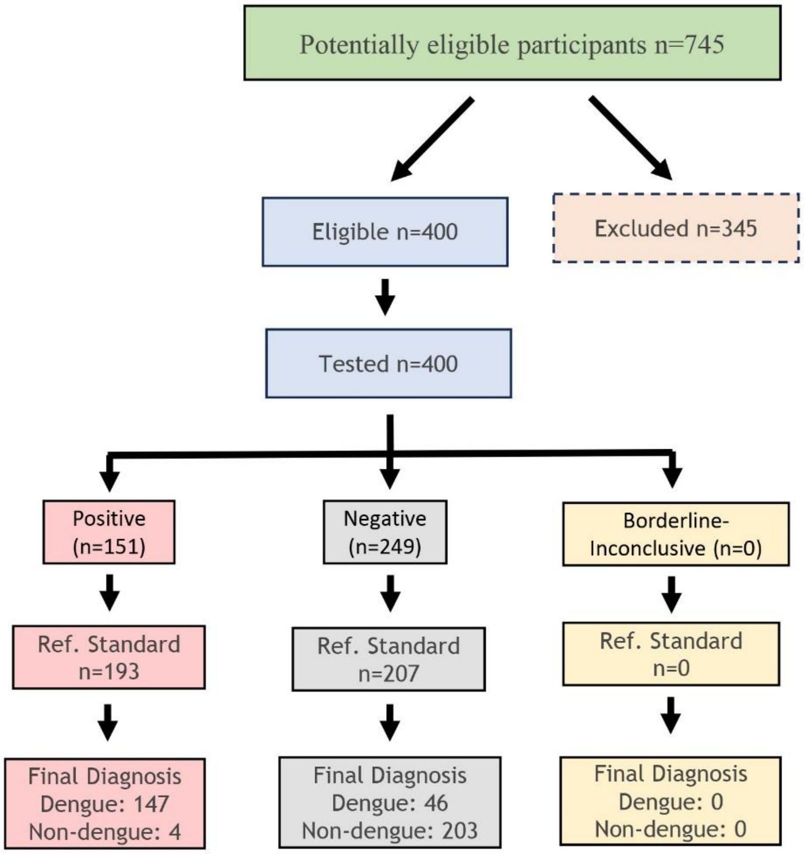

690

medRxiv preprint doi: https://doi.org/10.1101/2024.04.19.24306097; this version posted April 21, 2024. The copyright holder for this preprint (which was not certified by peer review) is the author/funder, who has granted medRxiv a license to display the preprint in perpetuity. This article is a US Government work. It is not subject to copyright under 17 USC 105 and is also made available for use under a CC0 license.

Figure S5. STARD flow diagram for CTK OnSite Dengue IgG Rapid Test R0065C-1.0 visual read

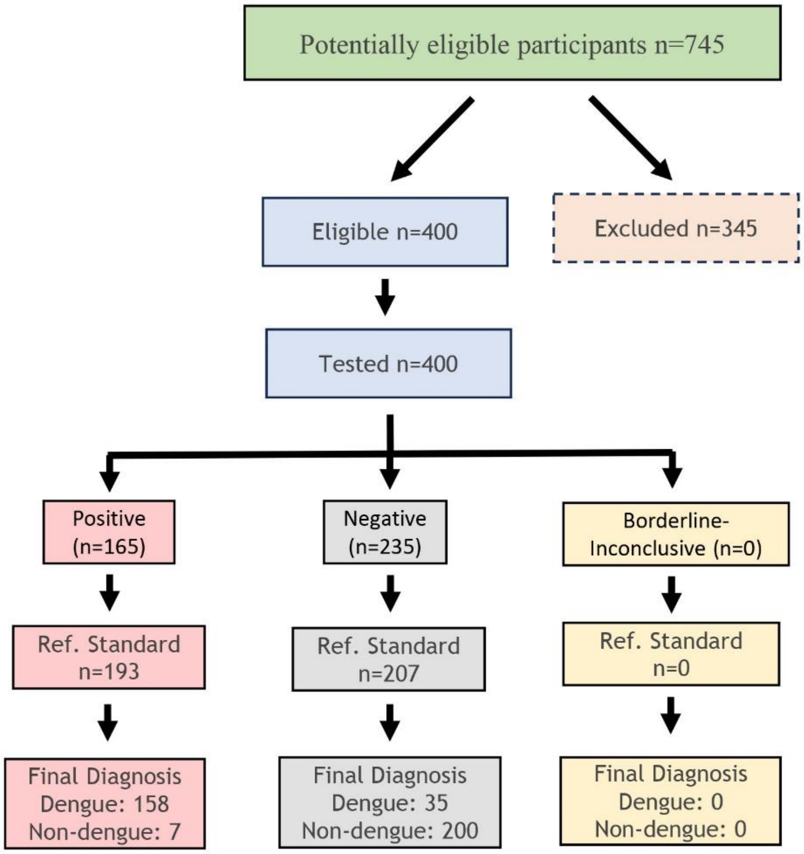

691

medRxiv preprint doi: https://doi.org/10.1101/2024.04.19.24306097; this version posted April 21, 2024. The copyright holder for this preprint (which was not certified by peer review) is the author/funder, who has granted medRxiv a license to display the preprint in perpetuity.
This article is a US Government work. It is not subject to copyright under 17 USC 105 and is also made available for use under a CC0 license.

Figure S6. STARD flow diagram for CTK OnSite Dengue IgG Rapid Test R0065C-1.0 equipment read

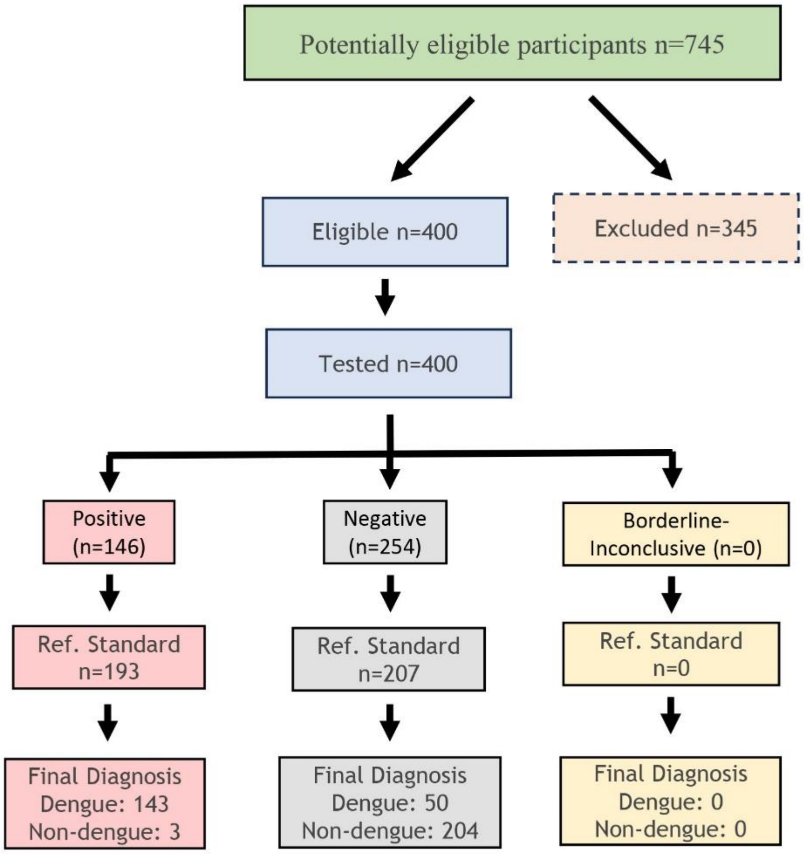

692

medRxiv preprint doi: https://doi.org/10.1101/2024.04.19.24306097; this version posted April 21, 2024. The copyright holder for this preprint (which was not certified by peer review) is the author/funder, who has granted medRxiv a license to display the preprint in perpetuity.
This article is a US Government work. It is not subject to copyright under 17 USC 105 and is also made available for use under a CC0 license.

Figure S7. STARD flow diagram for Euroimmun DENV NS1 Type 1-4 IgG ELISA + CTK OnSite Dengue IgG Rapid Test R0065C visual read

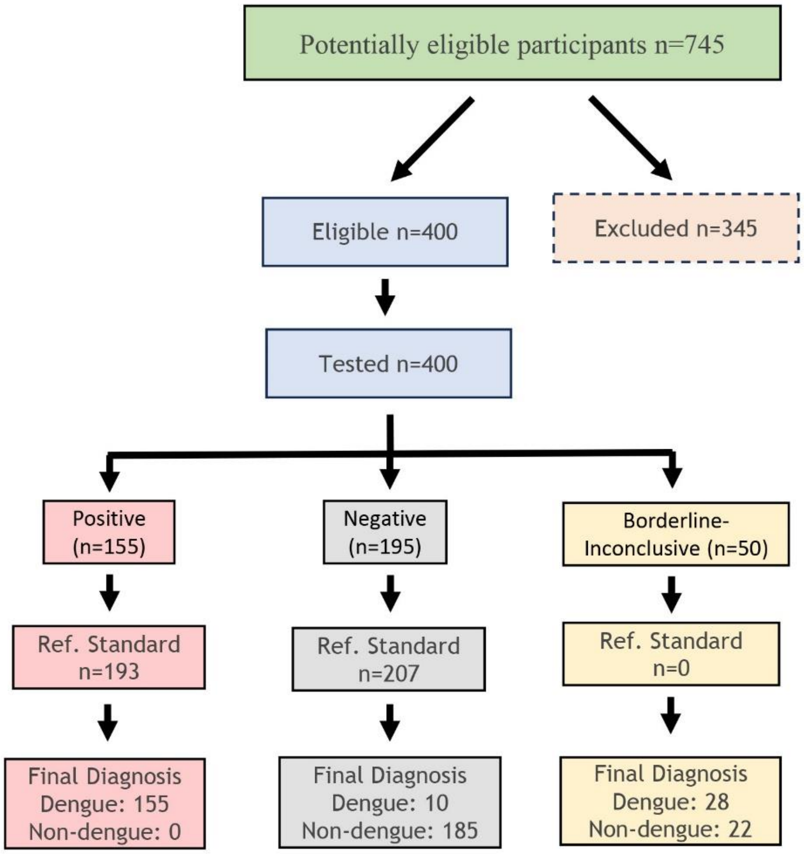

693

medRxiv preprint doi: https://doi.org/10.1101/2024.04.19.24306097; this version posted April 21, 2024. The copyright holder for this preprint (which was not certified by peer review) is the author/funder, who has granted medRxiv a license to display the preprint in perpetuity.
This article is a US Government work. It is not subject to copyright under 17 USC 105 and is also made available for use under a CC0 license.

Figure S8. STARD flow diagram for Euroimmun DENV NS1 Type 1-4 IgG ELISA + CTK OnSite Dengue IgG Rapid Test R0065C equipment read

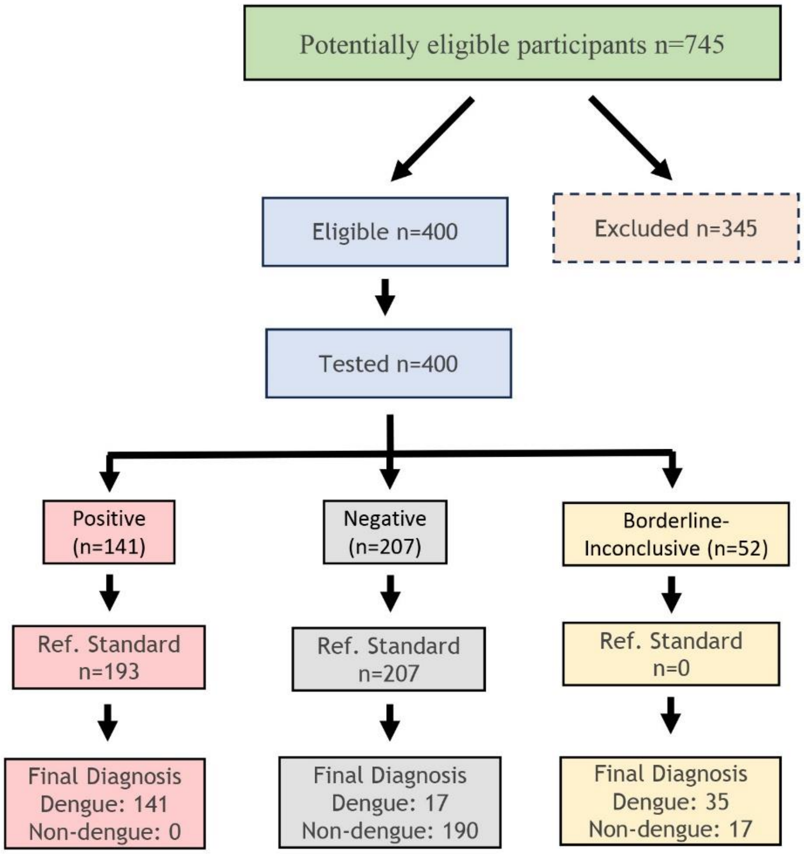

694

medRxiv preprint doi: https://doi.org/10.1101/2024.04.19.24306097; this version posted April 21, 2024. The copyright holder for this preprint (which was not certified by peer review) is the author/funder, who has granted medRxiv a license to display the preprint in perpetuity. This article is a US Government work. It is not subject to copyright under 17 USC 105 and is also made available for use under a CC0 license.

Figure S9. STARD flow diagram for Euroimmun DENV NS1 Type 1-4 IgG ELISA + CTK OnSite Dengue IgG Rapid Test R0065C-1.0 visual read

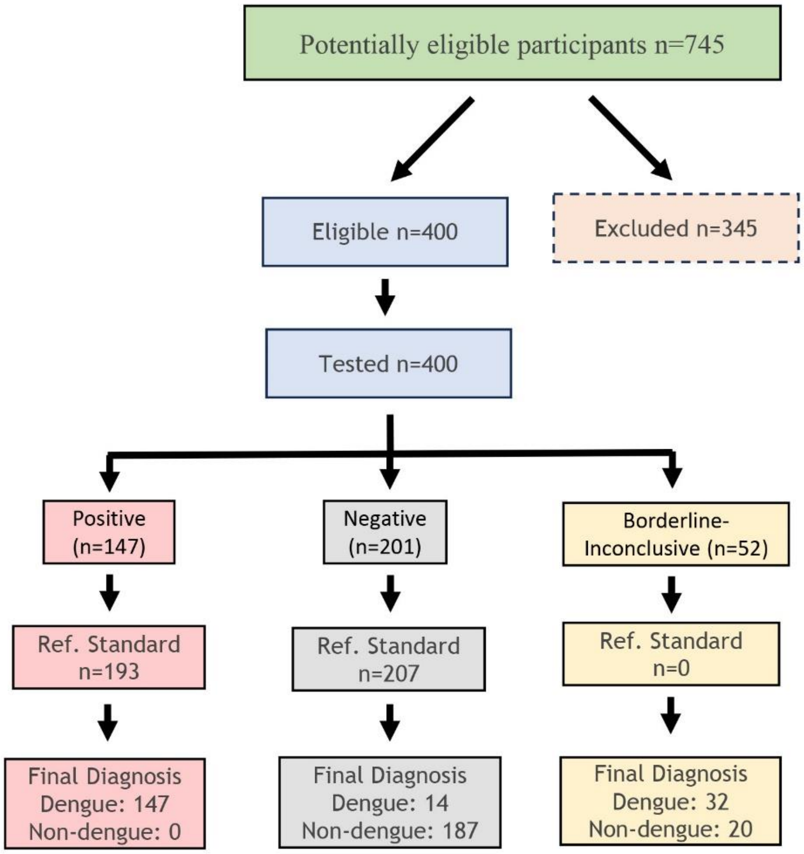

695

medRxiv preprint doi: https://doi.org/10.1101/2024.04.19.24306097; this version posted April 21, 2024. The copyright holder for this preprint
(which was not certified by peer review) is the author/funder, who has granted medRxiv a license to display the preprint in perpetuity.
This article is a US Government work. It is not subject to copyright under 17 USC 105 and is also made available
for use under a CC0 license.

Figure S10. STARD flow diagram for Euroimmun DENV NS1 Type 1-4 IgG ELISA + CTK OnSite Dengue IgG Rapid Test R0065C-1.0 equipment read

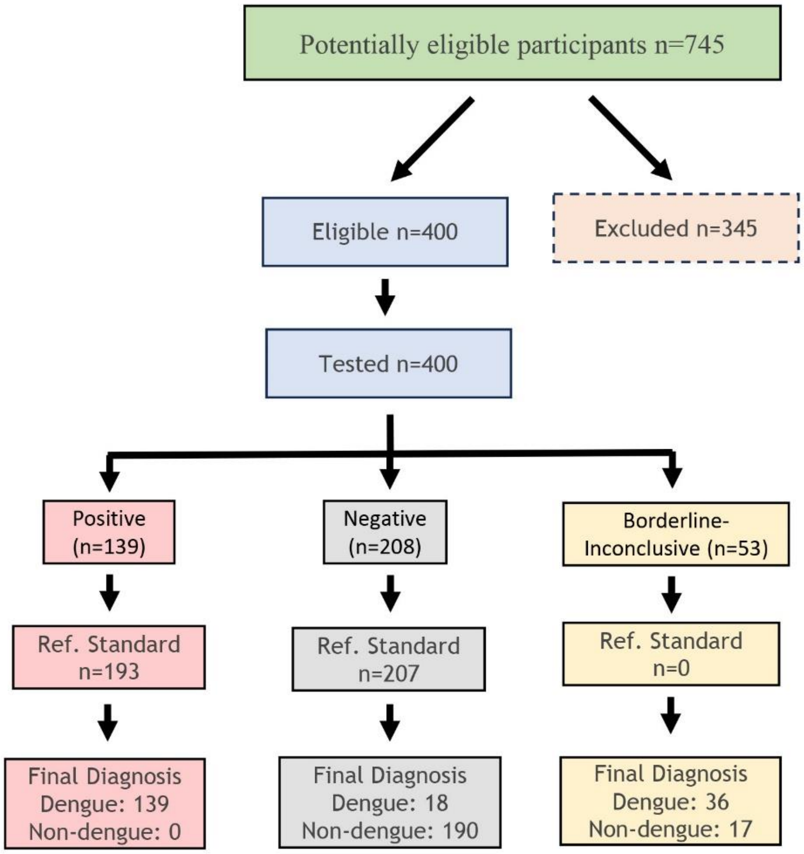

696

medRxiv preprint doi: https://doi.org/10.1101/2024.04.19.24306097; this version posted April 21, 2024. The copyright holder for this preprint (which was not certified by peer review) is the author/funder, who has granted medRxiv a license to display the preprint in perpetuity. This article is a US Government work. It is not subject to copyright under 17 USC 105 and is also made available for use under a CC0 license.

Figure S11. STARD flow diagram for Euroimmun DENV NS1 Type 1-4 IgG ELISA + CTK OnSite Dengue IgG Rapid Test R0065C visual read

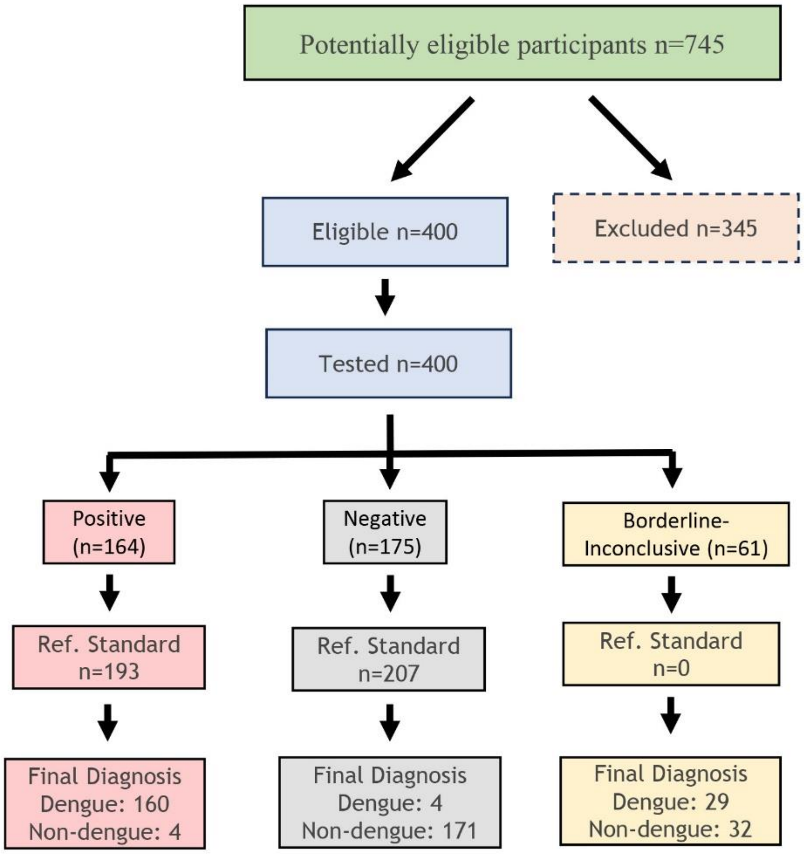

697

medRxiv preprint doi: https://doi.org/10.1101/2024.04.19.24306097; this version posted April 21, 2024. The copyright holder for this preprint (which was not certified by peer review) is the author/funder, who has granted medRxiv a license to display the preprint in perpetuity.
This article is a US Government work. It is not subject to copyright under 17 USC 105 and is also made available for use under a CC0 license.

Figure S12. STARD flow diagram for Euroimmun DENV NS1 Type 1-4 IgG ELISA + CTK OnSite Dengue IgG Rapid Test R0065C visual read

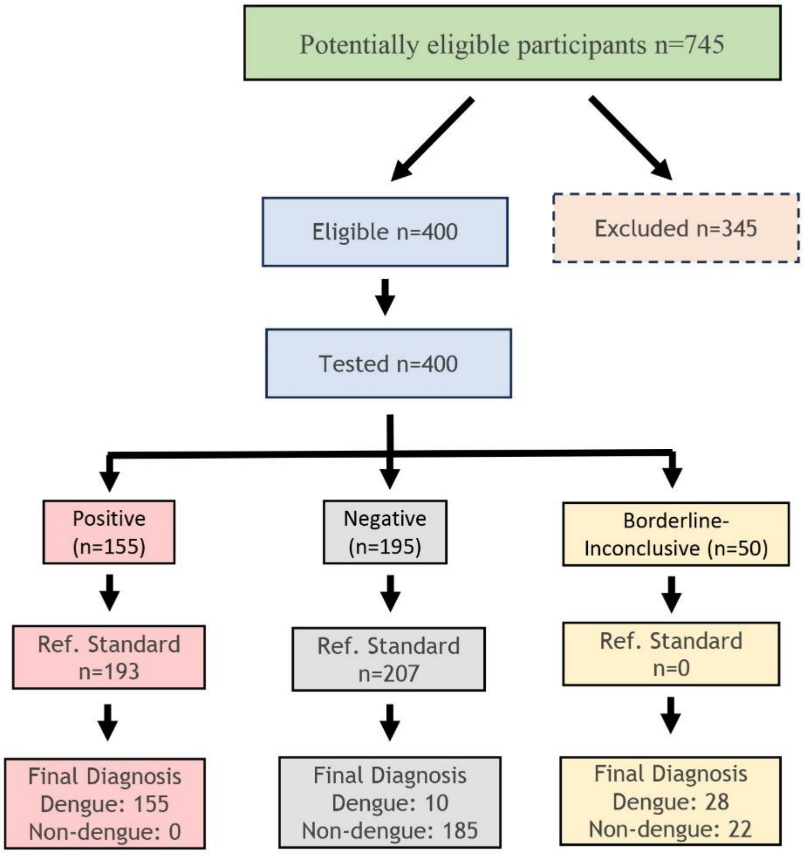

698

medRxiv preprint doi: https://doi.org/10.1101/2024.04.19.24306097; this version posted April 21, 2024. The copyright holder for this preprint (which was not certified by peer review) is the author/funder, who has granted medRxiv a license to display the preprint in perpetuity.
This article is a US Government work. It is not subject to copyright under 17 USC 105 and is also made available for use under a CC0 license.

STARD checklist

Section & Topic	No	Item	Reported on page #
**TITLE OR ABSTRACT**			
	**1**	Identification as a study of diagnostic accuracy using at least one measure of accuracy (such as sensitivity, specificity, predictive values, or AUC)	1
**ABSTRACT**			
	**2**	Structured summary of study design, methods, results, and conclusions (for specific guidance, see STARD for Abstracts)	3
**INTRODUCTION**			
	**3**	Scientific and clinical background, including the intended use and clinical role of the index test	4-6
	**4**	Study objectives and hypotheses	5-6
**METHODS**			
Study design	**5**	Whether data collection was planned before the index test and reference standard were performed (prospective study) or after (retrospective study)	7
Participants	**6**	Eligibility criteria	7
	**7**	On what basis potentially eligible participants were identified (such as symptoms, results from previous tests, inclusion in registry)	7
	**8**	Where and when potentially eligible participants were identified (setting, location, and dates)	7
	**9**	Whether participants formed a consecutive, random or convenience series	7
Test methods	**10a**	Index test, in sufficient detail to allow replication	13
	**10b**	Reference standard, in sufficient detail to allow replication	8-11
	**11**	Rationale for choosing the reference standard (if alternatives exist)	9
	**12a**	Definition of and rationale for test positivity cut-offs or result categories of the index test, distinguishing pre-specified from exploratory	14 (established by manufacturers)
	**12b**	Definition of and rationale for test positivity cut-offs or result categories of the reference standard, distinguishing pre-specified from exploratory	8
	**13a**	Whether clinical information and reference standard results were available to the performers/readers of the index test	14
	**13b**	Whether clinical information and index test results were available to the assessors of the reference standard	8
Analysis	**14**	Methods for estimating or comparing measures of diagnostic accuracy	15-16
	**15**	How indeterminate index test or reference standard results were handled	14
	**16**	How missing data on the index test and reference standard were handled	16
	**17**	Any analyses of variability in diagnostic accuracy, distinguishing pre-specified from exploratory	N/A
	**18**	Intended sample size and how it was determined	14
**RESULTS**			
Participants	**19**	Flow of participants, using a diagram	30-40
	**20**	Baseline demographic and clinical characteristics of participants	7
	**21a**	Distribution of severity of disease in those with the target condition	26, Table 3
	**21b**	Distribution of alternative diagnoses in those without the target condition	26, Table 3
	**22**	Time interval and any clinical interventions between index test and reference	14

medRxiv preprint doi: https://doi.org/10.1101/2024.04.19.24306097; this version posted April 21, 2024. The copyright holder for this preprint (which was not certified by peer review) is the author/funder, who has granted medRxiv a license to display the preprint in perpetuity.
This article is a US Government work. It is not subject to copyright under 17 USC 105 and is also made available for use under a CC0 license.

		standard	
Test results	**23**	Cross tabulation of the index test results (or their distribution) by the results of the reference standard	24, 26
	**24**	Estimates of diagnostic accuracy and their precision (such as 95% confidence intervals)	26, Table 3
	**25**	Any adverse events from performing the index test or the reference standard	12
**DISCUSSION**			
	**26**	Study limitations, including sources of potential bias, statistical uncertainty, and generalisability	21
	**27**	Implications for practice, including the intended use and clinical role of the index test	22
**OTHER INFORMATION**			
	**28**	Registration number and name of registry	
	**29**	Where the full study protocol can be accessed	
	**30**	Sources of funding and other support; role of funders	31
